# Integrated analysis of transcriptomic and metabolomic data reveals critical metabolic pathways involved in rotenoid biosynthesis in the medicinal plant *Mirabilis himalaica*

**DOI:** 10.1007/s00438-017-1409-y

**Published:** 2017-12-28

**Authors:** Li Gu, Zhong-yi Zhang, Hong Quan, Ming-jie Li, Fang-yu Zhao, Yuan-jiang Xu, Jiang Liu, Man Sai, Wei-lie Zheng, Xiao-zhong Lan

**Affiliations:** 1 Agricultural and Animal Husbandry College of Tibet University, Nyingchi, 860000 People’s Republic of China; 2 Key Laboratory of Ministry of Education for Genetics, Breeding and Multiple Utilization of Crops, College of Crop Science, Fujian Agriculture and Forestry University, Fuzhou, 350002 People’s Republic of China

**Keywords:** *Mirabilis himalaica* (Edgew.) Heimerl, Tibetan Plateau, Transcriptome, Metabolome, Rotenoid metabolic pathway

## Abstract

*Mirabilis himalaica* (Edgew.) Heimerl is among the most important genuine medicinal plants in Tibet. However, the biosynthesis mechanisms of the active compounds in this species are unclear, severely limiting its application. To clarify the molecular biosynthesis mechanism of the key representative active compounds, specifically rotenoid, which is of special medicinal value for *M. himalaica*, RNA sequencing and TOF-MS technologies were used to construct transcriptomic and metabolomic libraries from the roots, stems, and leaves of *M. himalaica* plants collected from their natural habitat. As a result, each of the transcriptomic libraries from the different tissues was sequenced, generating more than 10 Gb of clean data ultimately assembled into 147,142 unigenes. In the three tissues, metabolomic analysis identified 522 candidate compounds, of which 170 metabolites involved in 114 metabolic pathways were mapped to the KEGG. Of these genes, 61 encoding enzymes were identified to function at key steps of the pathways related to rotenoid biosynthesis, where 14 intermediate metabolites were also located. An integrated analysis of metabolic and transcriptomic data revealed that most of the intermediate metabolites and enzymes related to rotenoid biosynthesis were synthesized in the roots, stems and leaves of *M. himalaica*, which suggested that the use of non-medicinal tissues to extract compounds was feasible. In addition, the *CHS* and *CHI* genes were found to play important roles in rotenoid biosynthesis, especially, since *CHS* might be an important rate-limiting enzyme. This study provides a hypothetical basis for the screening of new active metabolites and the metabolic engineering of rotenoid in *M. himalaica*.

**Electronic supplementary material:**

The online version of this article (10.1007/s00438-017-1409-y) contains supplementary material, which is available to authorized users.

## Introduction

*Mirabilis himalaica* is a perennial herb belonging to the Nyctaginaceae family. As a Tibetan genuine medicinal herb, *M. himalaica* has a long history that can be traced back to 1300 years ago (Lan et al. 2014). *M. himalaica* mainly grows in Tibet, where the special ecological environment has provided its distinct medical value. *M. himalaica* has wide-ranging positive effects for disease treatment, including kidney warming, kidney nourishment, tissue regeneration, urination, and urinary calculus removal effects (Cai et al. 2013; Lan et al. 2014). The success of artificial cultivation technologies has completely changed the previous techniques, which relied only on wild medicinal resources. Recent studies on *M. himalaica* have mainly focused on the collection and evaluation of germplasm resources (Cai et al. 2013) and the establishment of artificial cultivation (Xu et al. 2013; Zhao et al. 2014; Guo et al. 2014). However, less research has been performed on the medicinal value of *M. himalaica* and the identification of its active medicinal compounds. Therefore, the quality of *M. himalaica* could have not been effectively evaluated. Moreover, insufficient data on the critical metabolites in *M. himalaica* has severely hindered the screening of its active medicinal compounds. On the other hand, many studies have shown that rotenoid, which exhibits remarkable anticancer effects, is among the active medicinal compounds in *M. himalaica* (Fan 2012; Linghu et al. 2014; Lan et al. 2016). However, the synthesis mechanism of rotenoid and its related metabolites in *M. himalaica* remains unclear because of a lack of available genetic information, especially at the molecular level.

Rotenoids are isoflavonoids and have important medicinal value, including marked anti-oxidant, antitumour, antibacterial, anti-inflammatory effects. Linghu et al. (2014) reported that rotenoid can block the S phase of the A549 cell cycle, which has important potential for cancer treatment (Linghu et al. 2014). Using rotenoid extracted from the roots of *Boerhavia diffusa*, Bairwa et al. (2013) found that rotenoid exhibits clear inhibitory effects on COX-1 and COX-2 activity (Bairwa et al. 2013). Aviello et al. (2011) proved that rotenoid inhibits the synthesis of thiobarbituric acid reactive species (TBARS) and ROS, enhances the activity of superoxide dismutase and reduces DNA damage caused by H_2_O_2_ (Aviello et al. 2011). Rotenoid exhibits various functions in disease treatment, but few studies have explored the rotenoid biosynthesis pathway. However, due to the development of transcriptomic and metabolomic technologies, many active medicinal compounds and related metabolic pathways have been rapidly identified in non-model plants. Therefore, it is now feasible to conduct detailed studies on the regulatory mechanisms and metabolic engineering of active medicinal compounds in these plants. Recent functional genomic studies on medicinal plants have achieved great progress. Transcriptomics have been widely used to reveal the biosynthetic pathways and regulatory mechanisms of key metabolites related to medicinal compounds in different medicinal plants (Wu et al. 2010, 2012; Rai et al. 2016). Moreover, the application of metabolomics to medicinal plants has significantly facilitated the identification of the metabolic pathways of the active medicinal compounds in the plants (Zhu et al. 2016; Akhatou et al. 2016). Furthermore, the integration of both transcriptomics and metabolomics has been widely used to reveal the biosynthetic mechanisms of key metabolic pathways, especially those in non-model plants (Ibáñez et al. 2014; Lin et al. 2015).

In this study, three transcriptomic libraries, one each of the roots, stems, and leaves of *M. himalaica*, were constructed via RNA-SEq. At the same time, the metabolites in the roots, stems, and leaves of *M. himalaica* were identified via UHPLC-QTOF-MS. Based on the transcriptomic and metabolomic information of *M. himalaica*, the metabolic pathways related to rotenoid biosynthesis in *M. himalaica* were revealed in detail by intermediary metabolites and the corresponding genes of enzymes. At the same time, the differential distribution of metabolites from the key secondary metabolic pathways was assessed from among the roots, stems and leaves. On basis of the above studies, the biosynthetic mechanism of rotenoid in *M. himalaica* was investigated in detail in this study. Our results provide important technical support for additional research on the metabolic regulation of rotenoid biosynthesis via the biological engineering and screening of other active medicinal compounds in *M. himalaica*.

## Materials and methods

### Sample collection

**Fig. 1:**
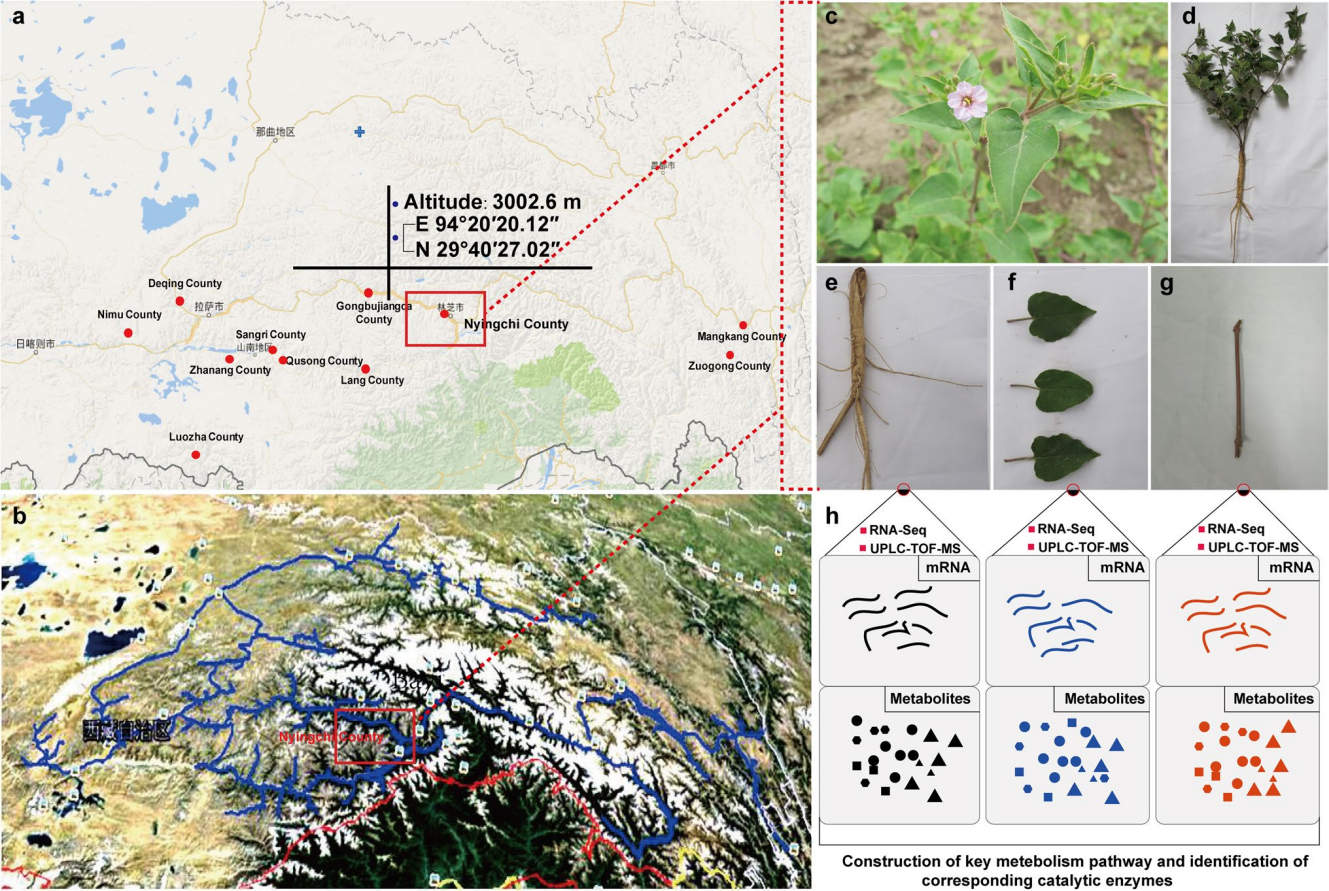
The typical ecological distribution of *M. himalaica* in Tibet. **a** The distribution area of *M. himalaica* in Tibet and sampling location. **b** Tibetan medicinal plant resource survey areas. **c**–**h** Collection of *M. himalaica* tissues for transcriptomic and metabolomic analysis and experimental procedures. The red dots represent the county-level distribution of *M. himalaica*, and the coordinates represent the geographic location of the experimental region of this study. The blue lines in b represent the recorded route during the resource survey

We sowed *M. himalaica* seeds at the Tibetan Medicinal Technology Demonstration Park of the Tibet Agriculture and Animal Husbandry College on May 28, 2016. A total of 3–4 *M. himalaica* seeds were sown into holes at a depth of 35 cm; the holes were located on a ridge with a width of 80 cm. After the seedlings emerged, only two seedlings exhibiting similar growth were retained per hole. We collected the roots, stems, and leaves of *M. himalaica* plants on September 15; the collected samples were wrapped in aluminium foil and then frozen in liquid nitrogen (Fig. 1). The samples were then analysed using transcriptomic and metabolomic approaches.

### Library preparation and Illumina HiSeq 2500 sequencing

The total RNA was isolated using an EASYspin Plus Plant RNA isolation kit (Aidlab, China). A 2100 Bioanalyzer was used to perform the total RNA quality control (QC) check. The qualified RNA samples were then digested by DNaseI (Takara, Japan) at 37 °C for 30 min and purified using Dynabeads^®^ Oligo (dT) 25 (Life, US). Afterwards, 100 ng of purified mRNA was used to establish a library using a NEBNext^®^ Ultra™ RNA Library Prep Kit for Illumina (NEB, US). First random primers and First Strand Synthesis Reaction Buffer (5×) was added to disrupt the mRNA, after which the mRNA was incubated at 94 °C for 15 min. The sample was chilled on ice immediately afterwards. The forward strand of the cDNA was synthesized by adding murine RNase inhibitor and ProtoScript II Reverse Transcriptase to the sample, while the reverse strand of the cDNA was synthesized by adding Second Strand Synthesis Reaction Buffer (10×) and Second Strand Synthesis Enzyme Mix to the sample. The samples were then purified by adding 1.8 × the volume of AMPure XP Beads (Agencourt, US), after which the bases were repaired and uracil was removed. Afterwards, 0.6 times the volume of AMPure XP Beads was added to the sample, the mixture was thoroughly mixed and then left to stand for 5 min, after which it was placed on a magnetic rack for 5 min. The supernatant was then removed, and 0.25 × AMPure XP Beads was added for purification. Ligated cDNA that ranged from 300 to 500 bp in size was subsequently retrieved. The samples were then amplified by 15 rounds of PCR and purified using an equivalent volume of AMPure XP Beads. The library was then retrieved and quantified using Qubit, 2% agarose electrophoresis, and a High-Sensitivity DNA chip to ensure the quality of the library. Finally, 10 ng of the library was used to generate clusters with cBot using a TruSeq PE Cluster Kit (Illumina, US) followed by two-way sequencing on an Illumina HiSeq™ 2500/MiSeq™. All the data have been registered in the NCBI Sequence Read Archive (SRA) database (https://trace.ncbi.nlm.nih.gov/Traces/sra/) under GenBank accession numbers SRR5908636, SRR5908643 and SRR5908646.

### De novo assembly and annotation

The raw paired-end reads were trimmed using Trimmomatic version 0.32 (Bolger et al. 2014), and QC was performed on the reads using FastQC version 0.10.0; the default parameters were used. The clean data from the 18 libraries were then used for RNA de novo assembly with Trinity version 2.06 (Grabherr et al. 2011). All the assembled transcripts were queried against the NCBI protein non-redundant (Nr), Swiss-Prot, and Kyoto Encyclopedia of Genes and Genomes (KEGG) databases using BLASTX to identify the proteins that had the highest sequence similarity with the given transcripts to retrieve their function annotations. A typical cut-off E-value of less than 1.0e^−5^ was used. InterProScan version 4.8 was used to obtain gene ontology (GO) annotations of unique assembled transcripts for describing biological processes, molecular functions, and cellular compounds (Zdobnov and Apweiler 2001). Metabolic pathway analysis was performed using the KEGG database (Kanehisa and Goto 2000).

### Differential expression analysis and candidate genes identification

To identify differentially expressed genes (DEGs) between two different samples, we calculated the expression level of each transcript in accordance with the fragments per kilobase of exon per million mapped reads (FPKM) method. SAMtools version 0.1.19 was used to quantify gene and isoform abundances (Li et al. 2009), and DEGseq version 1.20.0 was used for differential expression analysis (Wang et al. 2010). In addition, functional enrichment analysis, which included the use of the GO and KEGG databases, was performed to identify which DEGs were significantly enriched in GO terms and metabolic pathways at a Bonferroni-corrected *P* value ≤ 0.05 compared with those DEGs in the whole-transcriptome background. The GO functional enrichment and KEGG pathway analysis was carried out using GOseq (Young et al. 2010) and KOBAS software (Xie et al. 2011).

### qRT-PCR analysis

Total RNA (1 μg) from each sample was used to synthesize cDNA using a PrimeScript RT reagent kit (Takara, Japan). Quantitative real-time PCR (qRT-PCR) was conducted using SYBR premix Ex Taq (Takara, Japan). The analyses were carried out in accordance with the procedures described by Li et al. (Li et al. 2015), but with an annealing temperature of 62 °C. The 18S gene was selected as an internal control for normalizing the expression of the genes detected; the expression levels of this gene were more stable than those of the transcripts among the three organs (Lan 2015). The data were normalized on the basis of the 18S rRNA threshold cycle (Ct) value. The root samples were used as controls, and their normalized Ct values were set to 1. The relative expression of stem and leaf genes was calculated using the 2^−ΔΔCt^ method. The specific primer pairs used are listed in Supplemental Table S7. Three biological replicates were performed.

### Mass spectrum analysis and sample preparation

Three hundred-milligram samples were transferred to 2-mL Eppendorf (EP) tubes; each sample was added to three EP tubes. An amount of 1.2 mL of methanol, n-hexane, and ethyl acetate extracting solution was added to each tube, followed by the addition of 30 μL of adonitol (1 mg/mL stock in dH_2_O) as an internal standard. The mixture was then vortexed for 10 s, homogenized in a ball mill for 4 min at 45 Hz, ultrasonicated twice for 5 min each (incubated in ice water), incubated at − 20 °C for 1 h, and then centrifuged at 13,000 rpm at 4 °C for 15 min. Approximately 1 mL of the supernatant of each group was transferred to the same extraction solution, after which each solution was mixed for 10 s. A 0.3-mL aliquot of the supernatant from a different extraction solution was then transferred to a 1.5-mL EP tube; six samples were transferred per group. The extracts were dried in a vacuum concentrator in the absence of heat. Afterwards, 100 μL of an extraction solution [1:1 acetonitrile: water (*v*/*v*)] was added to reconstitute the samples. The samples were then vortexed for 30 s and sonicated for 10 min in a 4 °C water bath. The mixtures were subsequently centrifuged at 12,000 rpm at 4 °C for 15 min, after which the supernatant (60 μL) was transferred to a 2-mL LC/MS glass vial. A 10-μL aliquot from each sample was collected; these aliquots were then pooled as QC samples. A 60-μL aliquot of the supernatant was analysed via UHPLC-QTOF-MS, and six biological replicates were analysed.

### Mass spectrum parameter settings and data collection

The LC-MS/MS analyses were performed using an UHPLC system (1290, Agilent Technologies) with a UPLC bridged ethyl hybrid (BEH) amide column (1.7 μm, 2.1 mm × 100 mm, Waters) coupled to a TripleTOF 6600 (Q-TOF, AB Sciex) instrument (Ivanisevic et al. 2015). The mobile phase consisted of 25 mM NH_4_OAc and 25 mM NH_4_OH in water (pH = 9.75) (A), and acetonitrile (B) which was passed through the elution gradient as follows: 0 min, 85% B; 2 min, 75% B; 9 min, 0% B; 14 min, 0% B; 15 min, 85% B; and 20 min, 85% B, which was then delivered at 0.3 mL/min. The injection volume was 2 μL. The TripleTOF mass spectrometer was used because of its ability to acquire MS/MS spectra on an information-dependent basis (IDA) during LC/MS experiments. In this mode, the acquisition software (Analyst TF 1.7, AB Sciex) continuously evaluates the full scan-surveyed MS data, as it collects and triggers the acquisition of MS/MS spectra depending on the preselected criteria. In each cycle, 6 precursor ions whose intensities were greater than 100 were selected for fragmentation at a collision energy (CE) of 35 V (15 MS/MS events with a product ion accumulation time of 50 ms each). Electrospray ionization (ESI) source conditions were as follows: ion source gas 1 as 60; ion source gas 2 as 60; curtain gas as 30; source temperature of 550 °C; and ion spray voltage floating (ISVF) at 5500 V or 4500 V in positive or negative mode, respectively.

## Results

### Construction and functional analysis of the transcriptomic library of the roots, stems, and leaves of *M. himalaica*

Transcriptomic sequencing of the root, stem, and leaf libraries from *M. himalaica* generated 35,841,307, 40,177,727 and 45,070,198 raw reads, respectively. In all the three libraries, more than 98.54% of the sequences had a quality score greater than Q20 (Table 1), which indicated that the three libraries obtained high-quality raw reads. The raw data were subsequently filtered using Trimmomatic, resulting in 30,536,712, 34,528,903, and 39,472,174 clean reads obtained from the roots, stems, and leaves. The clean reads from the roots, stems, and leaves were assembled together using Trinity software. A total of 147,142 unigenes were obtained, which represented 94.1 Mb of transcription data; the average length of the unigenes was 650.20 bp, and the N50 was 842 bp (Supplemental Table S1; Supplemental Fig. S1).

**Table 1:** Statistics of the read data produced in different tissues

Sample	Mh.root	Mh.stem	Mh.leaf
Raw reads	35841307	40177727	45070198
Clean reads	30536712	34528903	39472174
Average length (bp)	2*150	2*150	2*150
Raw data	10.75G	12.05G	13.52G
Clean data	9.16G	10.36G	11.84G
Read 1 Q20	99.33%	99.33%	99.36%
Read 1 GC content	41.74%	42.11%	42.36%
Read 2 Q20	98.54%	98.62%	98.72%
Read 2 GC content	41.70%	42.08%	42.49%

**Fig. 2:**
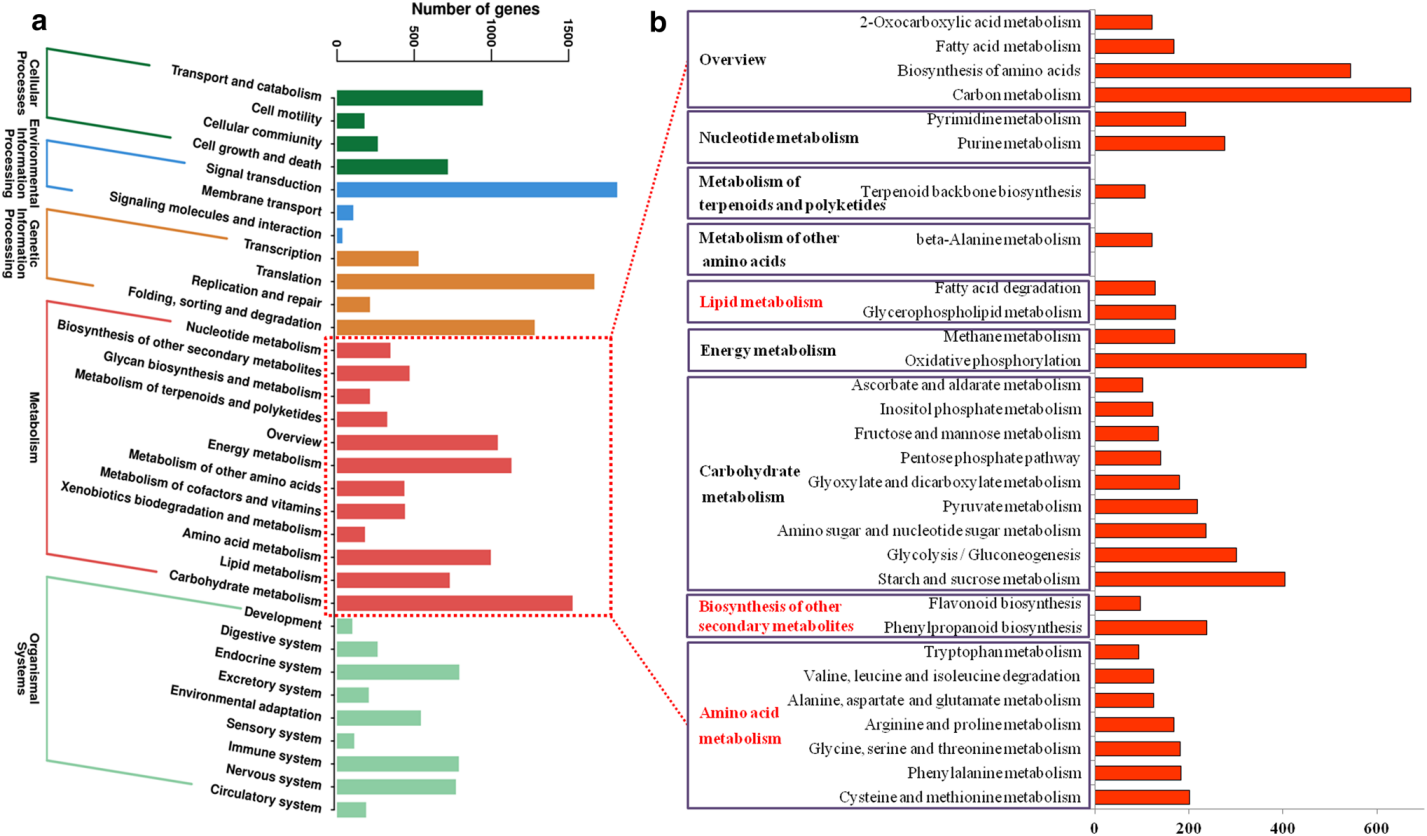
KEGG functional annotation of *M. himalaica* transcriptome assembly. **a** Functional annotation of *M. himalaica* transcriptome assembly in the KEGG pathways. **b** The top 30 metabolism class pathways are listed based on the number of assigned unigenes

To identify the functions of the *M. himalaica* unigenes, a homologous annotation of all the *M. himalaica* unigenes was performed based on information within the Nr, KEGG, and EuKaryotic Orthologous Groups (KOG) databases. A total of 36,940 sequences were annotated at least in a database (Supplemental Table S2), of which 35,763 unigenes had homologous sequences in the Nr database and 25,252 had homologous sequences in the SwissProt database. A total of 19,867 unigenes had KOG annotations; these unigenes were divided into 25 categories, including the metabolic categories of posttranslational modification (2423), signal transduction mechanisms (2421), and general function prediction (2011) (Supplemental Fig. S2). The GO database mainly involves biological processes, cellular compounds and molecular functions. In this study, the unigenes were divided into 40 GO terms (Supplemental Fig. S3). Among the biological processes, the GO terms with highest number of sequences were metabolic process (GO 0008152), cellular process (GO 0009987), and single-organism process (GO 0044699). Regarding the KEGG pathway annotations, 35,424 sequences were successfully annotated; these sequences were also identified in 32 KEGG subclasses (Fig. 2a). In all of the KEGG subclasses, compared with the other subclasses, the metabolism subclass had the most genes. The top 30 metabolic pathways are listed in Fig. 2b; of those pathways, amino acid metabolism involving cysteine and methionine metabolism (201) and phenylalanine metabolism (184) had the most mapped unigenes. Regarding the biosynthesis of other secondary metabolites, the pathways involving flavonoid biosynthesis (97) and phenylpropanoid biosynthesis (238) had the most mapped unigenes.

### Analysis the transcritomes of different *M. himalaica* tissues

**Fig. 3:**
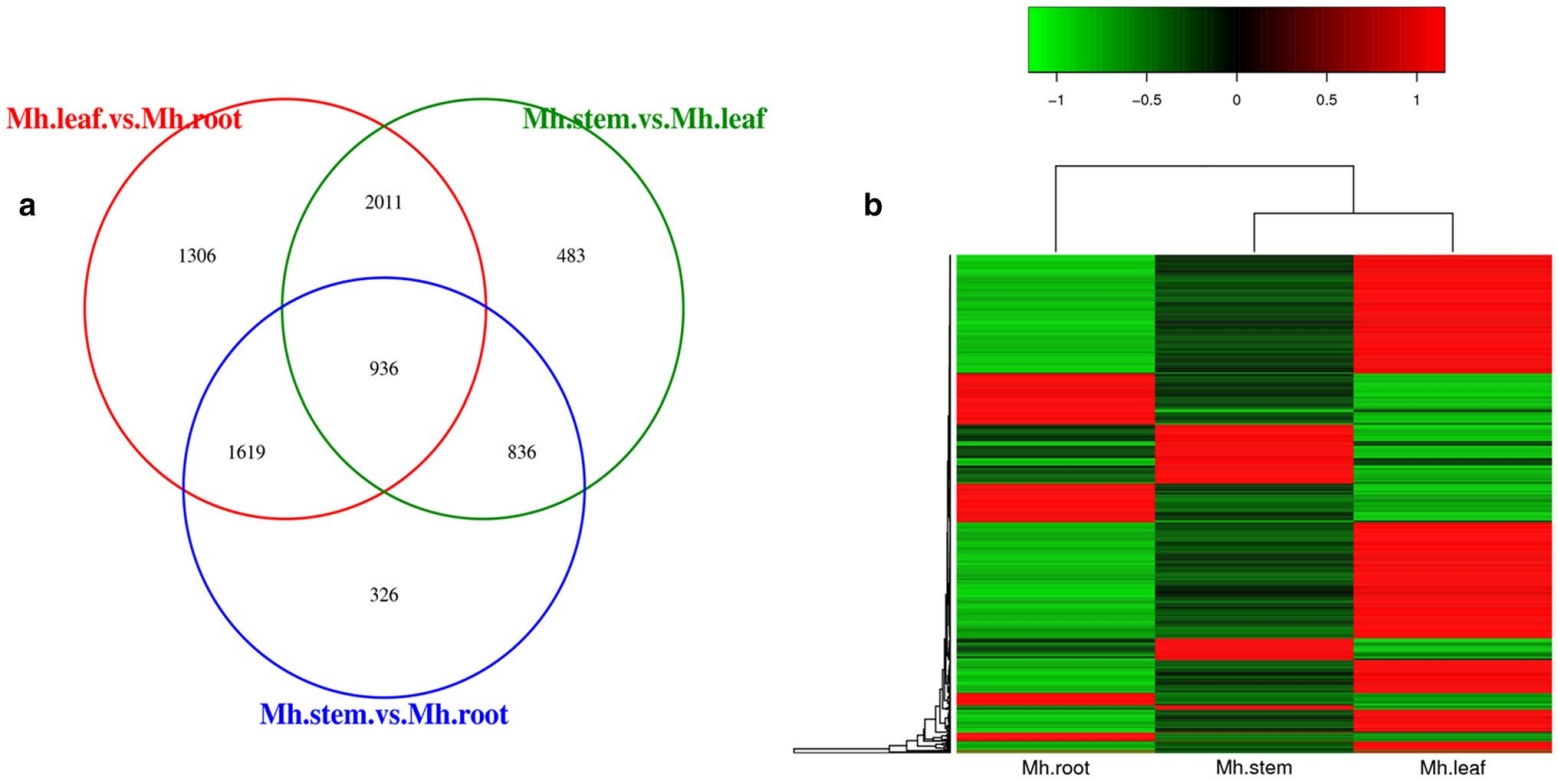
Statistics and analysis of DEGs among the different tissues in *M. himalaica*. **a** The numbers of DEGs among the different tissues. **b** Heatmap analysis of the DEGs among the different tissues. The analysis was based on the Nr, GO, and KEGG annotations for genes that had a |Fold change| > 2, an FDR ≤ 0.001, and at least one sample with an RPKM > 20

**Fig. 4:**
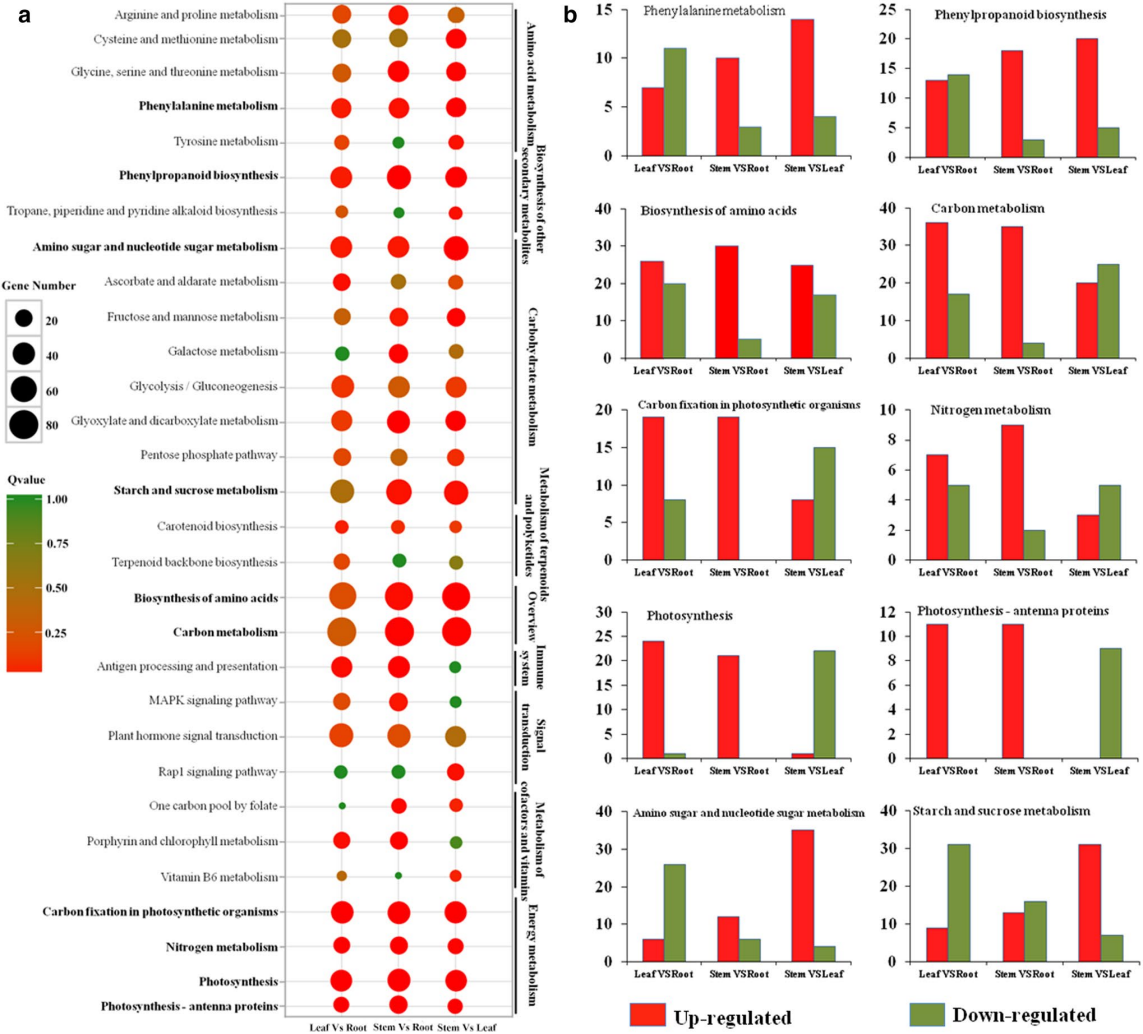
Top 20 enrichment pathways of DEGs, as analysed via pairwise comparisons between the different tissues

To reveal the gene expression patterns of the roots, stems, and leaves of *M. himalaica*, clean reads of the roots, stems, and leaves were mapped to the transcriptome using Bowtie (Langmead and Salzberg 2012). The resulting 24,804,679 (81.23%), 28,480,690 (82.48%), and 33,549,267 (84.99%) reads in the roots, stems, and leaves were mapped to the transcriptome of *M. himalaica* (Supplemental Table S3). The coverages of 34.59, 34.09 and 29.50% genes for the roots, stems and leaves, respectively, ranged from 90 to 100% (Supplemental Table S4). The gene expression patterns among the roots, stems, and leaves of *M. himalaica* were analysed using DEGs. The standard |fold change| > 2 and false discovery rate (FDR) < 0.001 parameters were used to identify the DEGs. A total of 5,872 genes, including 2544 upregulated genes and 3328 downregulated ones, were differentially expressed in the leaves and roots; 3717 genes, including 2326 upregulated genes and 1391 downregulated ones, were differentially expressed in the stems and roots; 4266 genes, including 2630 upregulated genes and 1636 downregulated ones, were differently expressed in the leaves and stems; and 936 genes were differently expressed in any two of the three tissues (Fig. 3a). The results of the hierarchical clustering analysis for the DEGs among different tissues showed that the expression patterns of stems and leaves were highly similar but differed between roots and stems (Fig. 3b). This finding suggested that stems and leaves might share similar biological processes. In addition, the results of the enrichment analysis of KEGG pathways for the DEGs among different tissues showed that numerous DEGs are involved in primary metabolic pathways between all the three tissues (Fig. 4a). At the same time, the enrichment pathways of the DEGs in different tissues reflected the preferential biological functions of the different tissues. For example, with respect to the photosynthesis, carbon metabolism, carbon fixation in photosynthetic organisms and nitrogen metabolic pathways, more genes were upregulated genes in the leaves than in the roots and stems, indicating that leaves play an important role in providing materials and energy for the growth and development of *M. himalaica* (Fig. 4b). In addition, in medicinal plants, the pathways related to secondary metabolism are the main subject of focus; this is especially true for phenylalanine metabolism and phenylpropanoid biosynthesis, both of which were clearly enriched between all the three tissues in the present study (Fig. 4a).

### Metabolomic analysis of the roots, stems, and leaves of *M. himalaica*

Candidate metabolic pathways in *M. himalaica* were identified via transcriptomic analysis. However, the validation of these key metabolic pathways in *M. himalaica* plants requires further confirmation by determining the presence of key metabolites. Metabolomic analysis of the roots, stems, and leaves of *M. himalaica* (six biological replicates each) was thus performed via UHPLC-QTOF-MS. As a result, 3287 and 2646 features were obtained in POS and NEG modes, respectively. The raw data were processed via missing value recoding and normalization followed by mass spectrum identification. A total of 419 peaks matched the secondary mass spectra in POS mode, and 180 peaks matched the secondary mass spectra in NEG mode. Based on the in-depth investigations of the metabolites of *M. himalaica*, the secondary mass spectrum data collected from both the ionization modes were integrated, and 522 metabolites were obtained (Supplemental Table S5).

To identify the general metabolic pathways of *M. himalaica*, 170 metabolites involved in 114 metabolic pathways were mapped to KEGG metabolic pathways (Supplemental Table S6). The pathways, in which the key intermediate metabolites could be clearly identified in the secondary mass spectra, commonly involved metabolic pathways, such as those of phenylalanine metabolism, tyrosine metabolism, valine, leucine and isoleucine biosynthesis. The results of the integrated analysis of the transcriptomic and metabolomic data for key metabolic pathways revealed that the transcript levels of catalytic enzyme genes are nearly consistent with abundance of their corresponding metabolites within the same pathway. For example, within phenylalanine metabolism, including its downstream flavonoid biosynthesis, isoflavonoid biosynthesis and phenylpropanoid biosynthesis branches, many intermediate metabolites were identified in the mass spectra, and at the same time, genes encoding catalytic enzymes were mapped to the pathways.

### Differential accumulation modes of metabolites among the different tissues of *M. himalaica*

To investigate the accumulation of metabolites, differences in the abundance of 170 metabolites among the different tissues of *M. himalaica* were studied, and the metabolic pathways of these metabolites were profiled. A total of 83 metabolites showed different abundances between the roots and leaves, 90 metabolites showed different abundances between the stems and roots, and 85 metabolites between the stems and leaves. An enrichment analysis of the pathways mediated by these metabolites was performed, and the top 20 significantly enriched pathways were selected for further analysis (Table 2). The results showed that the secondary metabolites that differed between the leaves and roots were involved mainly in isoflavonoid biosynthesis, flavone and flavonol biosynthesis, and flavonoid biosynthesis; the secondary metabolites that differed between the stems and roots were involved mainly in the isoflavonoid biosynthesis, phenylpropanoid biosynthesis, and flavonoid biosynthesis pathways; and those that differed between the stems and leaves were involved mainly in tyrosine metabolism, the degradation of aromatic compounds and glycerophospholipid metabolism. In contrast with the metabolomic information, the production of tyrosine, phenylalanine and tryptophan from prephenate and o-aminobenzoic acid in the shikimic acid pathway could be considered a key secondary metabolic pathway in *M. himalaica*. Moreover, this pathway is also a major source for generating aromatic metabolites such as ferulic acid, chlorogenic acid and rotenoid. In this study, the phenylalanine metabolic pathway and its related metabolic branches, such as flavonoid and isoflavonoid biosynthesis, were significantly enriched according to the KEGG analysis of differentially accumulated metabolites (Table 2). More importantly, the rotenoid specific to *M. himalaica* is among the most important metabolites produced from isoflavonoid biosynthesis. Therefore, the characterization of phenylalanine metabolism and its related metabolic branches was a critical step to further understand the biosynthetic mechanism of rotenoid and other core active medicinal compounds.

**Table 2:** Statistics of the numbers of differentially accumulated metabolites in the top 20 enrichment pathways among the different tissues

Pathway	Metabolites Number		Pathway	Metabolites Number	
	Leaf vs root	All		Stem vs root	All
Phenylalanine metabolism	5	10	Phenylalanine metabolism	7	10
Lysine degradation	3	5	Phenylalanine, tyrosine and tryptophan biosynthesis	4	4
Isoflavonoid biosynthesis	5	6	Isoflavonoid biosynthesis	6	6
Flavonoid biosynthesis	3	4	Flavonoid biosynthesis	4	4
Flavone and flavonol biosynthesis	3	3	Flavone and flavonol biosynthesis	3	3
Tyrosine metabolism	8	13	Tyrosine metabolism	8	13
Galactose metabolism	4	8	Phenylpropanoid biosynthesis	6	6
Propanoate metabolism	4	5	Betalain biosynthesis	3	3
Butanoate metabolism	3	4	Glucosinolate biosynthesis	6	9
Amino sugar and nucleotide sugar metabolism	4	8	Isoquinoline alkaloid biosynthesis	3	4
Protein digestion and absorption	11	20	Galactose metabolism	6	8
Carbohydrate digestion and absorption	3	4	Glycolysis/gluconeogenesis	3	3
Mineral absorption	7	12	Protein digestion and absorption	12	20
Phosphotransferase system (PTS)	4	5	Carbohydrate digestion and absorption	3	4
ABC transporters	14	24	Phosphotransferase system (PTS)	4	5
Nicotinate and nicotinamide metabolism	4	6	Proximal tubule bicarbonate reclamation	3	3
Pyrimidine metabolism	7	11	Nicotinate and nicotinamide metabolism	5	6
Purine metabolism	5	9	Pantothenate and CoA biosynthesis	3	4
Two-component system	5	6	cAMP signalling pathway	3	4
cAMP signalling pathway	3	4	Two-component system	4	6

### Differential expression and accumulation of genes and metabolites related to the phenylalanine metabolic pathway in different *M. himalaica* tissues

**Fig. 5:**
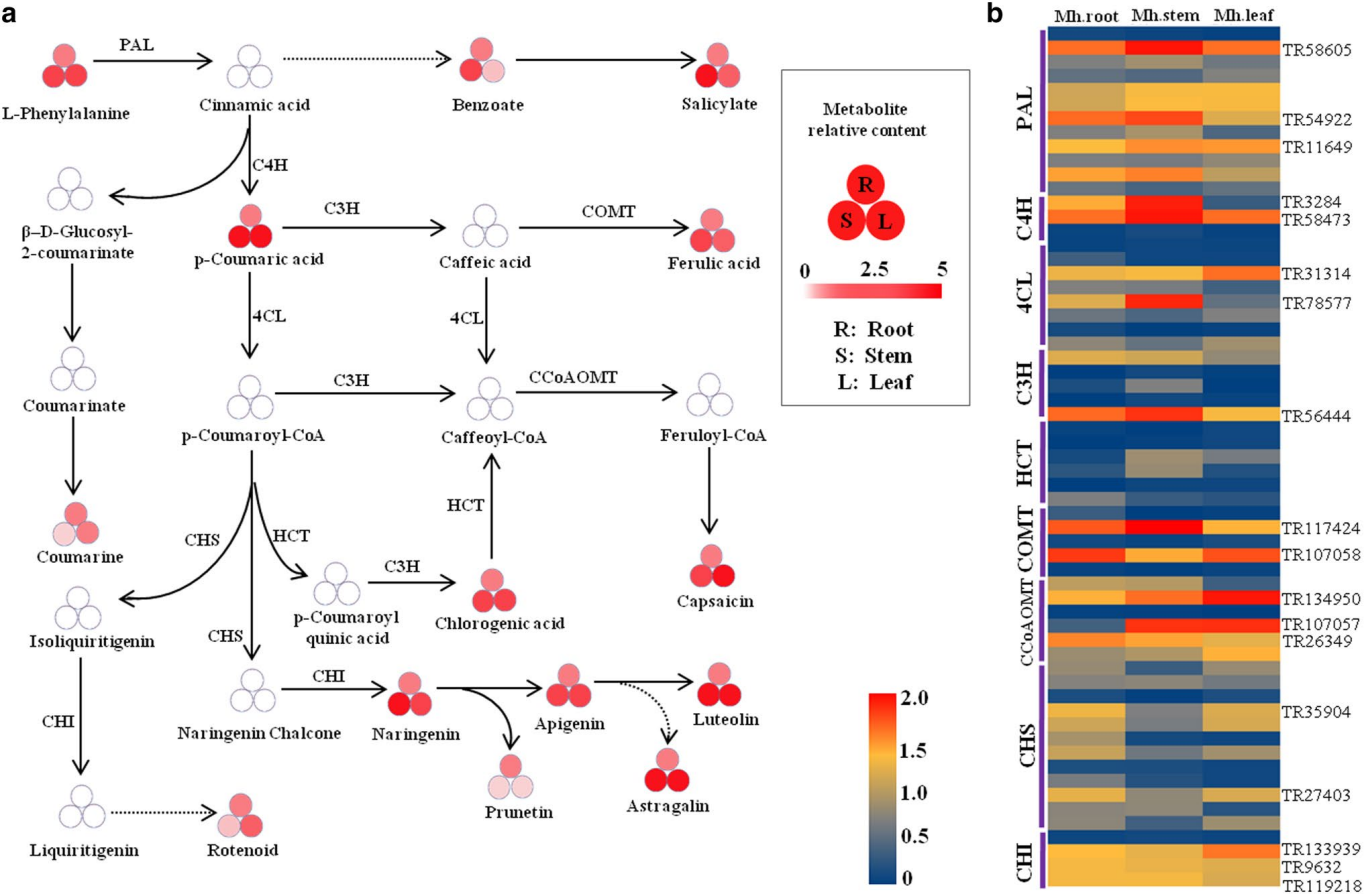
Different accumulation and expression patterns of metabolites and enzymes related to rotenoid biosynthesis in different tissues. **a** Differential accumulation of metabolites among the different tissues. The three red circles in a represent the different tissues, in which R represents the roots, S represents the stems and L represents the leaves. The colour scale represents the multiple of the relative content of each metabolite in the tissues compared to that in the roots. **b** Differential expression of the genes of key catalytic enzymes among the different tissues. The colour scale represents the transformed log_10_ (RPKM + 1) value of the unigenes in different tissues

To study the role of the phenylalanine metabolic pathway in the biosynthesis of active compounds in *M. himalaica*, a detailed, integrated analysis was performed on the transcript and metabolic levels involving phenylalanine metabolism, including phenylpropanoid biosynthesis, flavonoid biosynthesis, flavone and flavonol biosynthesis, and isoflavonoid biosynthesis. Fourteen metabolites, including L-phenylalanine, ferulic acid, capsaicin, chlorogenic acid, and rotenoid, were identified in phenylalanine metabolic pathway. At the same time, 61 genes encoding enzymes involved in phenylalanine metabolism were identified; these genes included *phenylalanine ammonia-lyase* (*PAL*), *cinnamate 4-hydroxylase* (*C4H*), *chalcone synthase* (*CHS*), and *chalcone isomerase* (*CHI*) among others. Based on these identified metabolites and genes encoding key enzymes, the phenylalanine metabolic pathway in *M. himalaica* was outlined (Fig. 5). p-Coumaroyl-CoA is an important metabolic substrate from which rotenoid is produced via two critical branches: one branch involves the synthesis of chlorogenic acid via catalysis by *HCT* and *C3H*; the other involves the production of the metabolites isoliquiritigenin and naringenin chalcone. The isoliquiritigenin is then converted to rotenoid via the *CHI* enzyme and other related steps, and naringenin chalcone is subsequently converted to other important active compounds, including naringenin, prunetin, apigenin, astragalin and luteolin. Moreover, the metabolites in the phenylalanine metabolic pathway exhibited different accumulation patterns among the *M. himalaica* tissues. For example, L-phenylalanine and prunetin accumulated mainly in the roots and less in the stems and leaves; benzoate, naringenin, and astragalin accumulated mainly in the stems; and capsaicin accumulated mainly in the leaves. Furthermore, rotenoid and coumarine were more abundant in the roots and leaves, whereas high levels of p-coumaric acid, chlorogenic acid, ferulic acid, and luteolin were detected in the stems and leaves (Fig. 5a). The tissues of *M. himalaica*, especially the leaves, which accumulate high amounts of rotenoid, could thus be considered potential raw materials for the screening of active medicinal compounds. In addition, the results of the transcriptomic analysis demonstrated that the key genes in the phenylalanine metabolic pathway were differentially expressed among different tissues. Moreover, the expression of the genes encoding the same catalytic enzymes showed clear differences among the different tissues (Fig. 5b). For example, both the TR117424 and TR107058 genes encode the *COMT* enzyme; TR11742 was upregulated in the stems and downregulated in the roots and leaves, whereas TR107058 was upregulated in the roots and leaves. When comparing the gene expression patterns and the intermediate metabolites in the phenylalanine metabolic pathway, the expression of most genes exhibited the same change trend as did the accumulation of the metabolites. For example, the expression of *CCoAOMT* (TR134950, TR107057), which catalyses the synthesis of capsaicin, was highest in the leaves but lowest in roots, and capsaicin also accumulated the most in the leaves. In addition, the expression trends of the *CHS* and *CHI* genes were consistent with accumulation of rotenoid among the different tissues. Specifically, the increased expression of the key enzyme *CHI* (TR133939), which catalyses rotenoid biosynthesis, in the roots and leaves was nearly consistent with the increased rotenoid accumulation in the same tissues.

### Quantitative gene expression and analysis

**Fig. 6:**
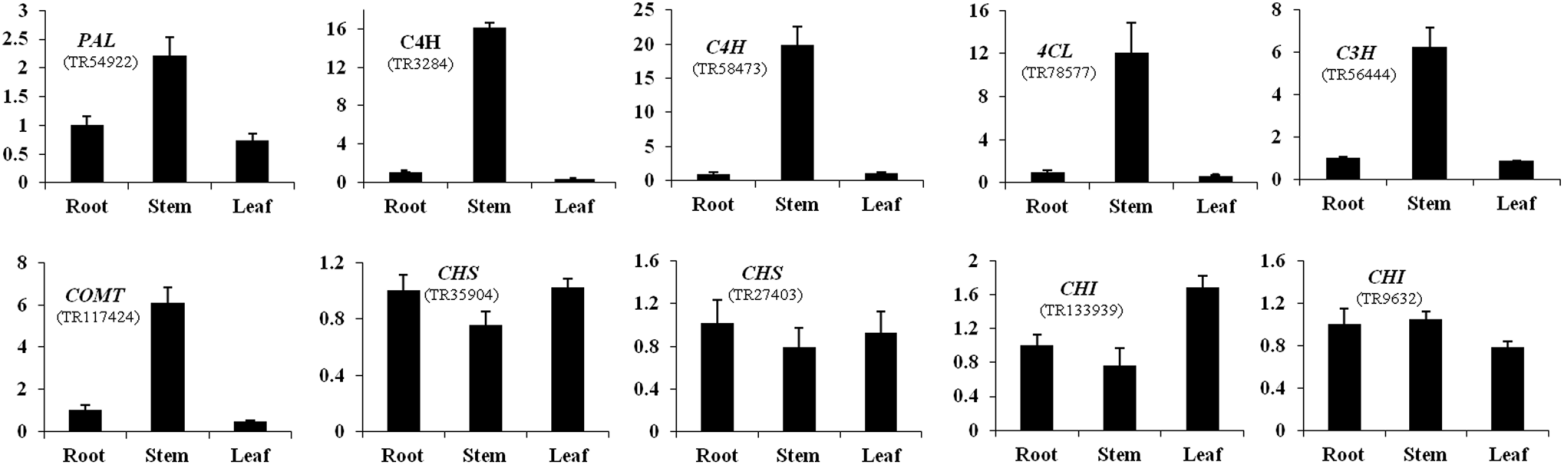
qRT-PCR verification of the genes of key enzymes in the phenylalanine metabolic pathway of *M. himalaica*

In this study, 10 genes were selected from the phenylalanine metabolic pathway to explore via qRT-PCR their expression profiles in different tissues (Fig. 6). The results showed that the *PAL* (TR58605, TR54922), *C4H* (TR3284, TR58473), *4CL* (TR78577), *C3H* (TR56444) and *COMT* (TR117424) genes exhibited the highest expression levels in the stems. However, the *CHS* and *CHI* genes, which are involved in rotenoid biosynthesis, were expressed less in the stems and more in the roots and leaves. In addition, 20 random genes of enzymes involved in the key metabolic pathways were also validated using qRT-PCR (Fig. S4). The expression patterns of all these genes in the different tissues quantified by qRT-PCR coincided with the results of the transcriptomic sequencing; small differences between *TyDC* (TR79530) and *FAH* (TR97815) were observed.

## Discussion

*Mirabilis himalaica* is a genuine Tibetan medicinal plant that grows in a plateau habitat. *Mirabilis himalaica* is widely distributed in areas of both Tibet and Sichuan. However, because of the complexity of the genetic background and lack of genetic information, investigations of the active medicinal compounds of *M. himalaica* and their related synthetic pathways have been hindered. To explore the active medicinal compounds and their synthetic mechanism in *M. himalaica*, transcriptomic libraries of the roots, stems, and leaves of *M. himalaica* were constructed by the use of high-throughput sequencing technology. At same time, the kinds of metabolites in the roots, stems, and leaves of *M. himalaica* were identified by UHPLC-QTOF-MS. A total of 10 Gb of clean data were obtained from the transcriptomic library of every different tissue; after being assembled, those data generated 147,142 unigenes. Using metabolomics technology, we identified 522 candidate compounds from *M. himalaica*, of which 170 were involved in 114 KEGG metabolic pathways. These obtained transcriptomic and metabolomic data provide an important data basis for further exploring these metabolic pathways of *M. himalaica*.

For *M. himalaica*, phenylalanine metabolism and its branches are important secondary metabolic pathways. In this study, 14 metabolites were identified in the phenylalanine metabolic pathway, which encompasses isoflavonoid biosynthesis, flavone and flavonol biosynthesis, flavonoid biosynthesis and phenylpropanoid biosynthesis. Most of these metabolites, including benzoic acid (Curini et al. 2012; Jeong et al. 2014), salicylic acid (Kong et al. 2008), chlorogenic acid (Jeong et al. 2014), p-coumaric acid (Lou et al. 2012), ferulic acid (Zhu et al. 2014), and naringenin (Rani et al. 2016) have antibacterial, anti-inflammatory, and anti-oxidative effects. Moreover, p-coumaric acid (Jaganathan et al. 2013), ferulic acid (Ou and Kwok 2004), naringenin (Harmon and Patel 2004), apigenin (King et al. 2010) and rotenoid (Linghu et al. 2014) exhibit anticancer properties. The important medicinal efficacy of these compounds is related to the potential accumulation of active medicinal compounds in *M. himalaica*. Interestingly, the results of the accumulation analysis of these metabolites in different tissues showed that the accumulation of many metabolites, such as benzoate, salicylate and rotenoid, increased in the stems and leaves; this finding was identical to that of Li et al. (Li et al. 2014). In general, the roots of *M. himalaica* have been considered as medicinal organs. The results of this study indicate that the stems and leaves could be potential raw materials for extracting useful compounds from *M. himalaica*. As such, these findings could help relieve the current situation that involves a lack of resources.

Rotenoid is the most common metabolite produced from phenylalanine metabolism in *M. himalaica* and exhibits important anti-inflammatory (Bairwa et al. 2013), anti-oxidative (Aviello et al. 2011), antiviral (Wangensteen et al. 2007), and anticancer therapeutic effects (Bortul et al. 2005; Lee et al. 2010; Linghu et al. 2014). Therefore, researchers should pay increased attention to the biosynthesis of rotenoid in *M. himalaica*. In *M. himalaica*, rotenoid is primarily derived from the isoflavonoid biosynthesis pathway (Lan et al. 2016). However, the detailed steps of rotenoid biosynthesis remain largely unknown. Isoflavonoid biosynthesis and its related metabolic pathways, such as those of flavone biosynthesis, flavonol biosynthesis and flavonoid biosynthesis, are important downstream pathways for phenylalanine metabolism. These metabolic pathways are particularly important for exploring rotenoid biosynthesis. However, the lack of genetic information has delayed the study of these metabolic pathways in *M. himalaica*. The results of this study proved that two branches that use p-coumaroyl-CoA as a metabolic substrate are closely related to rotenoid biosynthesis: one branch could generate chlorogenic acid via catalysis by *HCT* and *C3H*; the other one produced the metabolites isoliquiritigenin and naringenin chalcone. Isoliquiritigenin is ultimately converted to rotenoid via *CHI* and a series of steps thereafter, and naringenin chalcone is converted to key compounds such as naringenin, prunetin, apigenin, astragalin and luteolin (Fig. 5). *CHS* and *CHI* play important roles during the upstream catalysis of rotenoid, but some critical nodes remain largely unknown (Lan et al. 2016). In the present study, the combined analysis of the transcriptomic and metabolic profiles of *M. himalaica* indicated that some metabolites downstream of naringenin chalcone metabolism were produced in types and amounts more than those involved in rotenoid biosynthesis from isoliquiritigenin. Notably, in both the p-coumaroyl-CoA to naringenin chalcone or p-coumaroyl-CoA to isoliquiritigenin processes, both *CHS* and *CHI* are irreplaceable enzymes. Therefore, we primarily thought that naringenin chalcone synthesis might compete with rotenoid synthesis. Furthermore, the expression trend of the *CHS* family genes was highly consistent with the accumulation of rotenoid in different tissues; rotenoid accumulation was higher in the roots and leaves than in the stem. However, the expression of the majority of enzyme genes involved in phenylalanine metabolism, such as *PAL, C4H* and *4CL*, was higher in the stems (Fig. 5b). *CHS* may thus be an important rate-limiting enzyme involved in the process of rotenoid biosynthesis. Moreover, the expression trend of *CHI* (TR133939) was consistent with the accumulation pattern of rotenoid. Therefore, the *CHI* genes play an important role in the molecular synthesis of rotenoid (Lan et al. 2016).

Through the use of an integrated analysis of transcriptomics and metabolomics, the metabolic pathways of *M. himalaica* were clearly outlined, which provides a deep understanding of the biosynthetic mechanism of the active medicinal compounds in *M. himalaica*. The unravelling of the biosynthetic mechanism of rotenoid at the molecular level especially provides a theoretical basis and reference for further metabolic engineering of rotenoid.

## Conclusions

This study first investigated the genuine Tibetan medicinal plant *M. himalaica* via high-throughput sequencing and metabolomic analysis. A total of 147,142 unigenes and 522 metabolites were identified from the transcriptomic and metabolomic information. Moreover, the different accumulation and expression patterns of the metabolites and enzymes in the associated metabolic pathways of rotenoid biosynthesis were analysed in different tissues. The transcriptomic and metabolic data especially led to a clearer understanding of the synthetic mechanism of rotenoid: *CHS* and *CHI* genes play important roles, and *CHS* might be considered an important rate-limiting enzyme in the process of rotenone biosynthesis. This result provides a theoretical basis and reference for further metabolic engineering of rotenoid. In addition, the transcriptomic and metabolomic information lays the foundation for the screening of new active metabolites and for future studies of the biosynthetic mechanisms of those metabolites in *M. himalaica*.

## Electronic supplementary material

Below is the link to the electronic supplementary material.

**Fig S1** Distribution of unigene lengths in *M. himalaica* (XLS 706 KB)

**Fig S2** KOG classification of transcribed CD-containing sequences in *M. himalaica* (TIF 1721 KB)

**Fig S3** GO classification of transcribed CD-containing sequences in *M. himalaica* (TIF 1803 KB)

**Fig S4** qRT-PCR verification of the gene expression of key catalytic enzymes in *M. himalaica* (TIF 131 KB)

Supplementary material 5 (XLS 20 KB)

Supplementary material 6 (XLS 12246 KB)

Supplementary material 7 (XLS 19 KB)

Supplementary material 8 (XLS 22 KB)

Supplementary material 9 (XLS 291 KB)

Supplementary material 10 (XLS 52 KB)

Supplementary material 11 (XLS 26 KB)

## Abbreviations

DEGs: Differentially expressed genes
RPKM: Per million mapped sequence reads
KEGG: Kyoto encyclopaedia of genes and genomes
GO: Gene ontology
MARS: MA-plot-based method with random sampling model
FDR: False discovery rate
POS: Positive ions
NEG: Negative ions
CHS: Chalcone synthase
CHI: Chalcone isomerase
PAL: Phenylalanine ammonia-lyase
C4H: Cinnamate 4-hydroxylase
4CL: 4-Coumarate-CoA ligase
C3H: Cinnamate 3-hydroxylase
HCT: Shikimate O-hydroxycinnamoyl transferase
COMT: Caffeic acid 3-O-methyltransferase
CCoAOMT: Caffeoyl-CoA O-methyltransferase
