# Supplementary figures and images for: Integrated analysis of transcriptomic and metabolomic data reveals critical metabolic pathways involved in rotenoid biosynthesis in the medicinal plant Mirabilis himalaica
Source: Mol Genet Genomics. 2017 Dec 28;293(3):635–47. doi: 10.1007/s00438-017-1409-y (PMC5948277; doi:10.1007/s00438-017-1409-y)

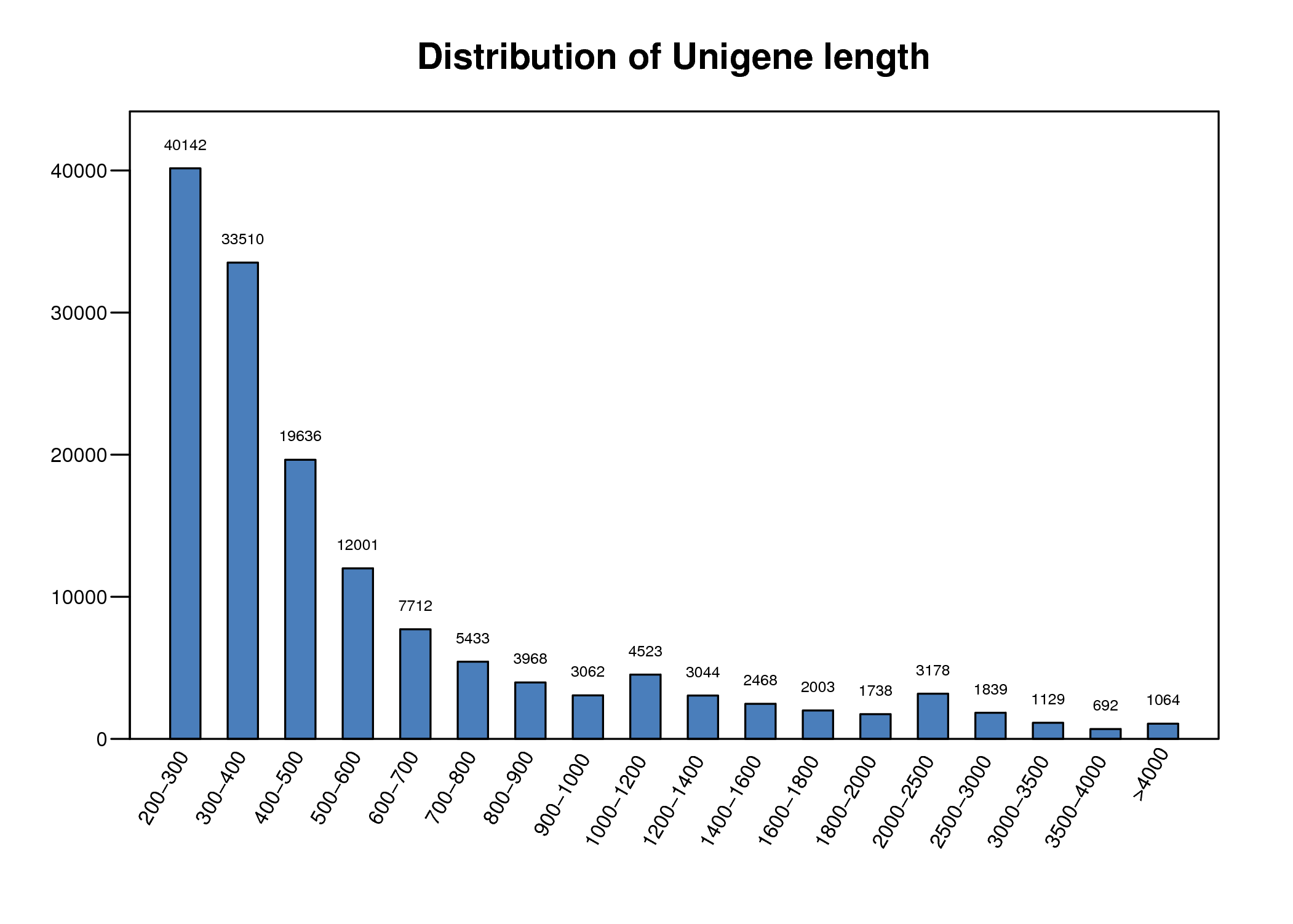

Supplement: Supplementary file 1 — Fig S1 Distribution of unigene lengths in M. himalaica (XLS 706 KB) [file 438_2017_1409_MOESM1_ESM.xls]

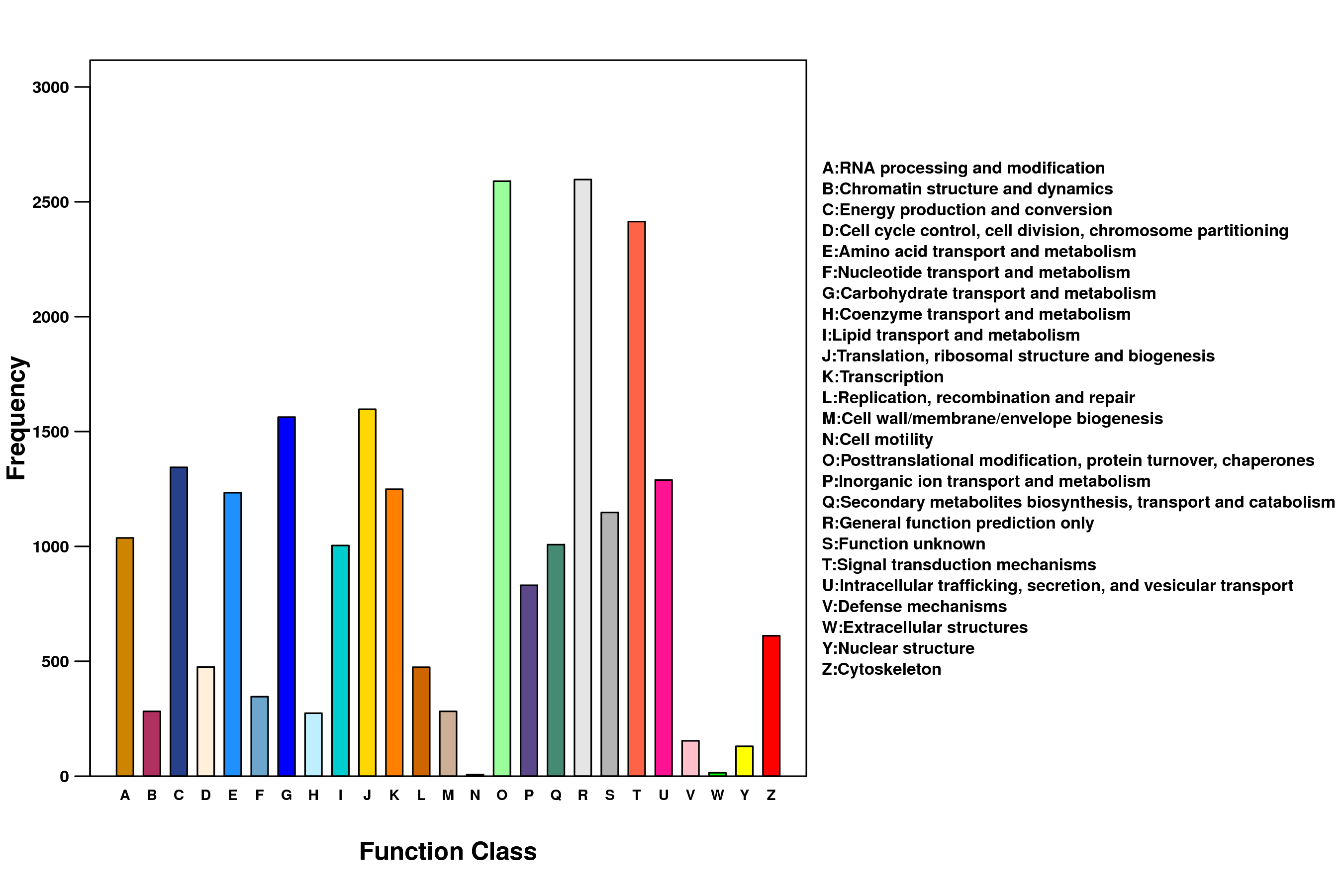

Supplement: Supplementary file 2 — Fig S2 KOG classification of transcribed CD-containing sequences in M. himalaica (TIF 1721 KB) [file 438_2017_1409_MOESM2_ESM.tif]

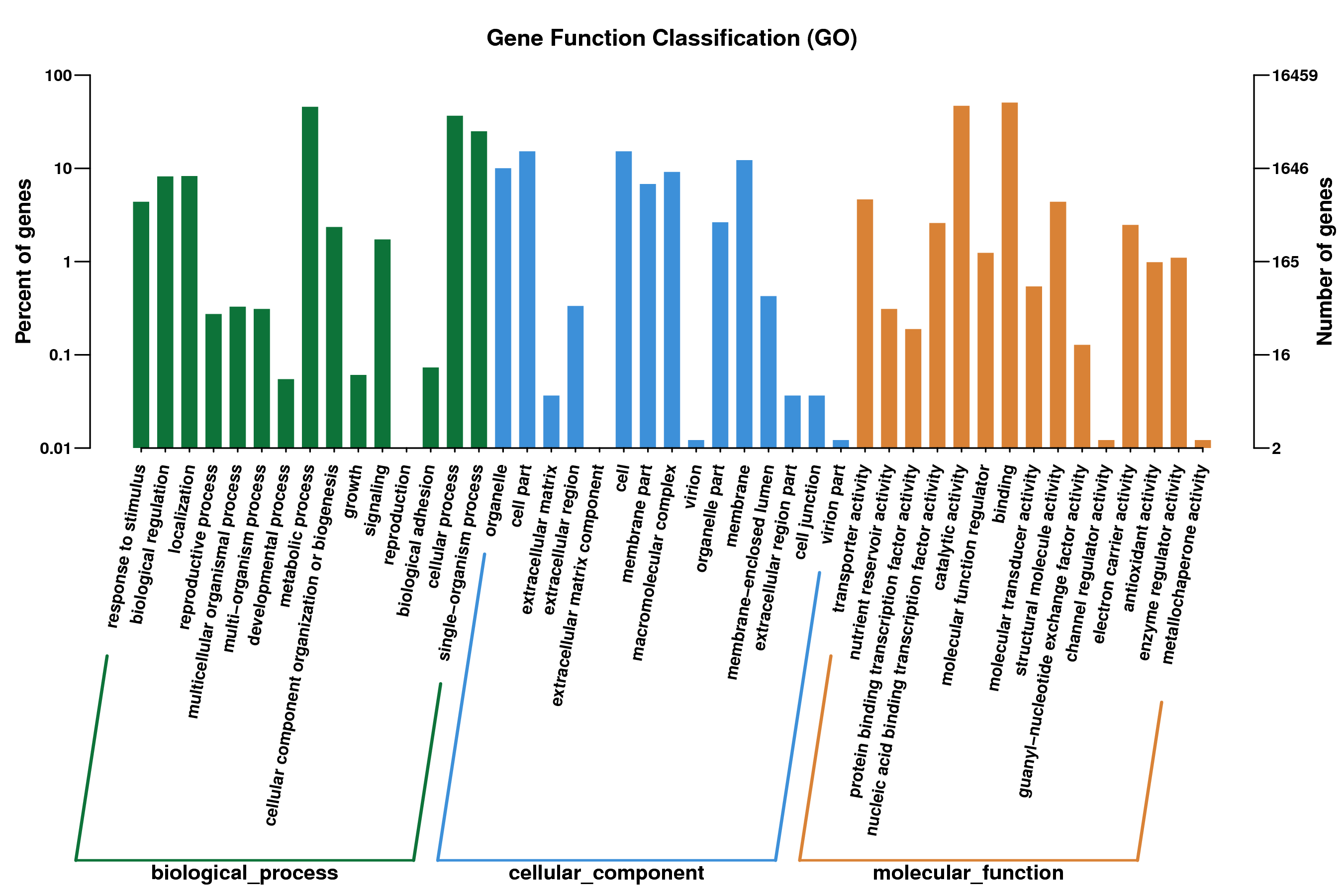

Supplement: Supplementary file 3 — Fig S3 GO classification of transcribed CD-containing sequences in M. himalaica (TIF 1803 KB) [file 438_2017_1409_MOESM3_ESM.tif]

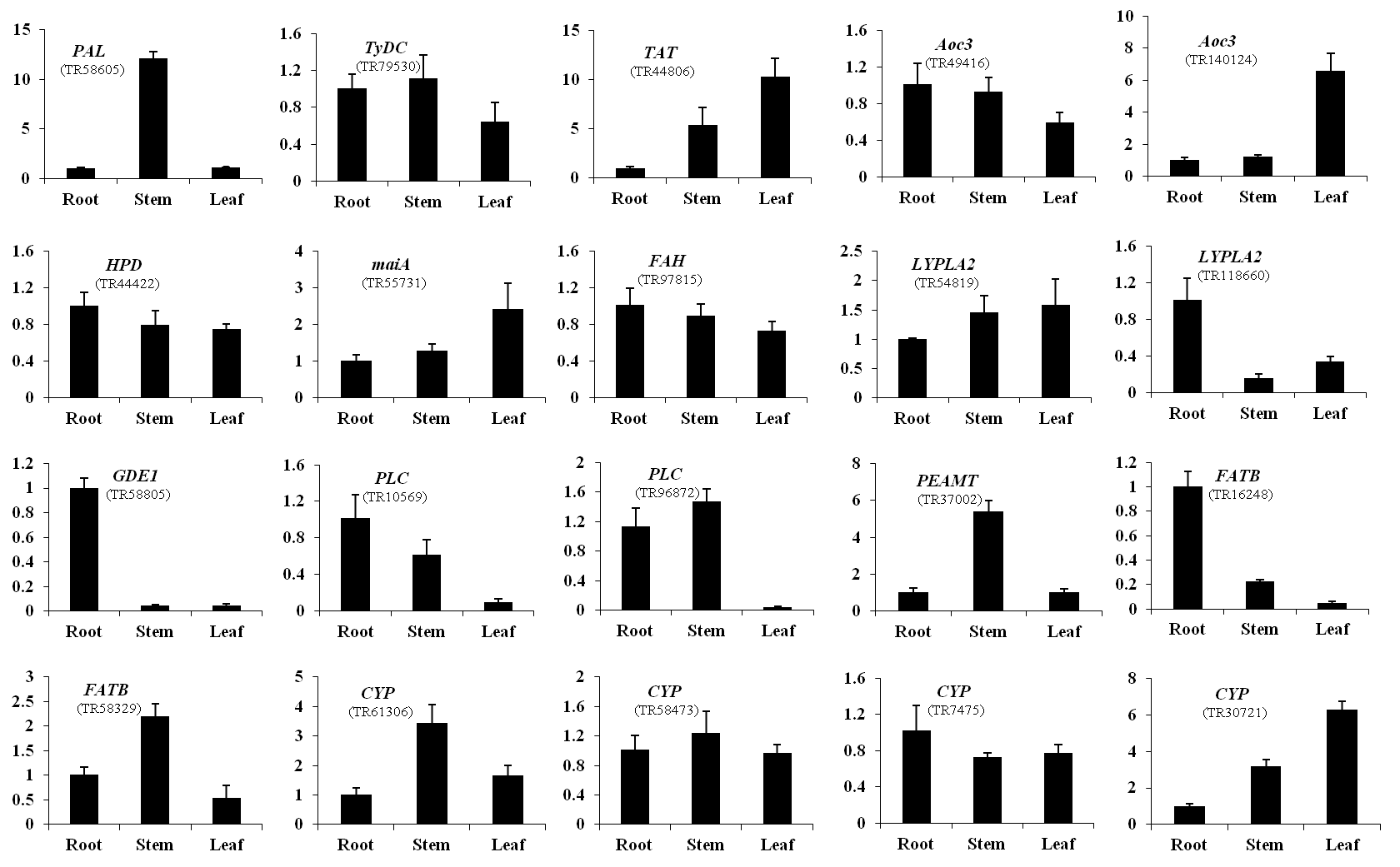

Supplement: Supplementary file 4 — Fig S4 qRT-PCR verification of the gene expression of key catalytic enzymes in M. himalaica (TIF 131 KB) [file 438_2017_1409_MOESM4_ESM.tif]
